# In Situ and Home Care Nasopharyngeal Intubation Improves Respiratory Condition and Prevents Surgical Procedures in Early Infancy of Severe Cases of Robin Sequence

**DOI:** 10.1155/2015/608905

**Published:** 2015-07-26

**Authors:** Isabel Cristina Drago Marquezini Salmen, Ilza Lazarini Marques

**Affiliations:** Department of Pediatrics, Hospital for Rehabilitation of Craniofacial Anomalies, University of São Paulo, Rua Sílvio Marchioni 3-20, 17043900 Bauru, SP, Brazil

## Abstract

*Aim*. To evaluate the clinical outcome of infants with Robin Sequence (RS) and severe respiratory obstruction managed with nasopharyngeal intubation (NPI). *Methods*. This prospective study was conducted with 107 infants with RS admitted to the Hospital for Craniofacial Anomalies of the University of São Paulo (HRAC-USP), from July 2003 to June 2010, diagnosed with severe RS and treated with NPI. The infants were followed up for the first year of life. Clinical findings, morbidity, and mortality were recorded. *Results*. Of the 223 infants with RS admitted to the hospital in the period studied, 149 were diagnosed with severe respiratory distress and 107 (71.81%) matched all the inclusion criteria. Of those, 78 (73%) presented Isolated Robin Sequence and 29 (27%) presented other syndromes or anomalies associated with RS. NPI treatment lasted an average of 57 days and the mean hospitalization time was 18 days. Although all infants presented feeding difficulties, 85% were fed orally and only 15% underwent gastrostomy. Morbidity was 14% and no deaths occurred. *Conclusions*. The children treated with the RS treatment protocol adopted at the HRAC-USP had improved respiratory and feeding difficulties, required a shorter hospitalization time, and presented low morbidity and mortality during the first year of life. The general outcome prevented surgical procedures in early infancy.

## 1. Introduction

Previously known as the Pierre Robin syndrome, Robin Sequence (RS) affects one in 8,500 live human births [1]. It is characterized by shortened mandible (micrognathia) and posteriorly placed tongue (glossoptosis). Cleft palate may also be present, but it is not observed in all cases [2]. Micrognathia seems to be the trigger for a cascade of events leading to tongue displacement, cleft palate, and respiratory distress, feeding difficulties, and consequent poor growth (hence the term “sequence”) [3]. RS can occur as an isolated anomaly (IRS) or in association with other syndromes or anomalies (SRS) [4, 5].

Infants with RS present a challenge to pediatricians and other specialists because of their increased risk of airway obstruction and resultant hypoxia, cor pulmonale, failure to thrive, and cerebral impairment. As the infants grow, airway obstruction improves as the mandible grows and the coordination of the parapharyngeal muscles improves in conjunction with voluntary tongue control [6]. The goal of the initial treatment is to minimize any airway obstruction to prevent hypoxia and to promote normal neurologic development and include prone positioning [3], nasopharyngeal intubation (NPI) [7–10], glossopexy [11, 12], mandibular distraction osteogenesis [13, 14], and tracheostomy [15]. However, because of the lack of studies based on a large number of children with RS in a single center, much controversy remains about the use of both nonsurgical and surgical intervention strategies to manage respiratory obstruction in RS patients.

One of the current treatment strategies, the NPI procedure, helps the tongue to move forward, freeing the airway and allowing the child to breathe through the nasopharyngeal tube. Previous studies report having used NPI to relieve airway obstruction successfully in RS infants [16, 17] and to prevent the use of surgical procedures during early infancy of RS patients [6]. These reports suggest that natural growth may lead to resolution of airway obstruction without the use of unnecessary surgical interventions.

In 2003, the Hospital for Craniofacial Anomalies of the University of São Paulo (HRAC-USP) established a new RS treatment protocol replacing glossopexy with NPI for the treatment of severe cases of children with RS. Over the years, the HRAC-USP has gained a large experience with NPI management of RS [8–10]. Here, we present the current RS treatment protocol employed at the HRAC-USP and analyze the evolution of a large series of severe cases of children with RS treated exclusively with NPI. We recorded the duration of NPI use, the frequency of gastrostomy, the age at the time of referral, associated syndromes, clinical symptoms, type of respiratory obstruction, and clinical complications during the first year of life and mortality. The data collected was used for the longitudinal and prospective analysis reported here. The results revealed herein may help clinicians make decisions regarding the need for surgical intervention strategies to manage airway obstruction in infants with severe RS.

## 2. Methods

### 2.1. Patients

Two hundred and twenty-three infants with RS were admitted to the HRAC-USP from July 2003 to June 2010. The infants were diagnosed as mild, moderate, or severe cases through objective airway assessment carried out using continuous oxygen saturation and through clinical observation by experienced staff. Severe cases presented recurrent crises of pallor and/or cyanosis and/or apnea, intercostal and supraclavicular retractions, oxygen saturation < 90% measured by continuous pulse oximetry with an oxygen requirement to improve this condition, and severe feeding difficulties for which feeding tubes were necessary. Mild cases had little respiratory difficulty without intercostal retraction or retraction of the furcula, O_2_ saturation measured by continuous pulse oximetry equal to or higher than 90.0%, and few feeding difficulties (feeding exclusively by the oral route); moderate cases had intercostal retraction or retraction of the furcula without cyanosis, apnea, or pallor, satO_2_ greater than 90.0%, and important feeding difficulties (feeding by a nasogastric tube) [18]. Of 223 infants, 74 presented mild or moderate symptoms and were managed with prone positioning and 149 infants presented severe symptoms. Of 149 severe cases, 107 were treated exclusively with NPI and 42 underwent tracheostomy. Only children diagnosed as severe RS, submitted to the RS management protocol adopted at the HRAC-USP and treated exclusively with NPI, were included in the study. Thus, of the infants hospitalized during this period, 107 children met the inclusion criteria.

### 2.2. RS Treatment Protocol at the HRAC-USP

Nasopharyngoscopy was performed in all children during the first days of hospitalization and the type of respiratory obstruction was classified according to Sher et al. [19]: type 1: the tongue is retro positioned and touches the posterior pharynx wall; type 2: the tongue presses the palate against the pharynx wall; type 3: there is a medial contraction of the pharynx and the pharynx is the cause of obstruction, while the tongue does not touch the pharynx wall; and type 4: the contraction of the pharynx is sphincteric.

All exams were performed by the same professional, a plastic surgeon with extensive experience in nasopharyngoscopy, and nasopharyngoscopy took place in the operating theater, in a room appropriate for this purpose. The infants were examined in horizontal dorsal decubitus without head flexion, awake, and without any type of sedation. An Olympus nasopharyngoscope (Tokyo, Japan) for infants with an INF P3 fiber (OTV-SC video camera system with a DSR 20 MD digital videocassette) was introduced through the right nostril with topical lidocaine chlorohydrate 2% (gel). All evaluations were performed by the same professional.

**Figure 1:**
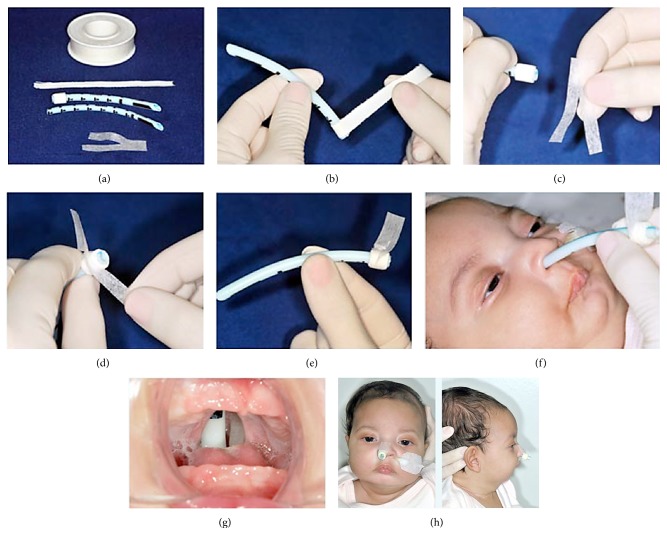
Nasopharyngeal intubation. (a) The Portex silicone tube and tape for marking the tube; (b) tube marked with a tape at the 7.5 cm level for positioning of the tube 7.5 cm inside and 1 cm outside the nostril; (c) the 5 cm micropore, partially divided in half; (d) the micropore tape fixed on the tube through divided parts; (e) the tube ready for introduction into the nostril; (f) the tube being introduced into the nostril; (g) a view of a tube through the cleft palate; (h) infant with PRS and nasopharyngeal intubation.

NPI was performed in infants with type 1 and 2 respiratory obstructions who displayed severe respiratory symptoms. NPI consists of a whitish Portex silicone tube of 3.0–3.5 cm that is introduced 7 to 8 cm into the nostril, cut 1 cm out of the nostril, and fixed with micropore tape. The tube is placed just above the epiglottis to allow the air to flow through it (Figure 1).

Improvement of respiratory discomfort with NPI was considered to have occurred when O_2_ saturation, measured by continuous pulse oximetry, was maintained above 90% in ambient air (with no oxygen requirement) during 24 hours; when the respiratory effort was reduced (reduction of pallor and cyanosis crises, of intercostal and furcula retraction, and of inspiratory noise characteristic of glossoptosis observed by pulmonary auscultation); when it was possible to stimulate oral feeding and the child became comfortable with NPI, without accumulation of secretions and saliva in the oral and/or nasal cavity and/or in the tube for NPI. After the infant's respiratory discomfort had improved the parents were trained to manage NPI. Patients are only discharged after a period of monitoring with the NPI in situ and when clinical staff was sure of caretakers competence.

After discharge from the hospital, return visits to the hospital were scheduled every 15 days during its continuous use. At each return the infant was hospitalized for 24 hours for observation, and the definitive removal of NPI was performed only when, in the absence of the NPI, O_2_ saturation remained above 90% in ambient air for 24 hours with no onset or worsening of respiratory discomfort. Otherwise, NPI was maintained until reevaluation on subsequent return visits.

Clinical observations and assessment were performed by multidisciplinary staff (nurses, pediatricians).

After decannulation, children are followed up at three-month intervals until the end of the first year of life.

Children with types 1 or 2 who did not improve with NPI were submitted to tracheostomy. Because types 3 and 4 are not considered RS but Robin complex (as the tongue is not the cause of respiratory obstruction), all children diagnosed as these types and with severe symptoms were submitted to tracheostomy to release the airways due to the severity of the respiratory obstruction [6].

All children were submitted to feeding facilitating techniques (FFTs) to improve oral feeding [20] and were given a hypercaloric diet. FFTs include sucking on a pacifier, receiving a massage to interiorize and relax the tongue, improving lip closure and preventing oral escape of the food, using a long and soft nipple with a hole enlarged to 1 mm, and using thickened milk. To thicken the milk, corn-based industrially modified flour is added to the concentration of approximately 3%, until a thickened liquid consistency is achieved. The hypercaloric diet consists of a milk formula supplemented with 5 to 7% glucose polymers and 3 to 5% medium-chain triglycerides with essential fatty acids.

Children were considered ready for exclusively oral feeding when they were able to ingest 70% of the milk volume recommended for their age, in less than 30 minutes, without choking and/or a reduction in oxygen saturation.

## 3. Ethics

This study was approved by the Research Ethics Committee of HRAC-USP (SVAPEPE-CEP number 265/2011). All patients' parents or legal guardians signed a written informed consent.

## 4. Results

One hundred seven infants, 52 boys (48.6%) and 55 girls (51.4%), were followed up during the first year of life. Mean age at the time of referral was 31.7 +/− 29 days (ranging from one day to five months). Most patients (62/107) were younger than one month of age at referral. Seventy-eight children (73%) presented IRS and 29 (27%) presented SRS (Table 1).

All infants presented severe symptoms at the time of admission, and 56 infants (52%) were admitted to the intensive care unit. Ninety-five (89%) infants presented upper airway obstruction type 1 and two infants (11%) presented type 2 obstruction (Table 2).

The mean time of NPI use was 57.4 +/− 37.6 days (ranging from 1 to 173, median 54) and the mean hospitalization time for children treated with NPI was 18 days (ranging from 2 to 57, median 16). All patients were followed up until the NPI was no longer required and there were no nasal injuries, no untoward incidences at home, and no problems related to the use of NPI.

Most patients had cleft palate (104/107). All infants presented feeding difficulties; 91 infants (85%) were fed orally and gastrostomy was performed in 16 infants (15%). Gastrostomy was more frequent in infants with SRS (31%) than with IRS (9%) (Table 3).

The most frequent complication was pneumonia. Morbidity was 14% and no deaths occurred (Table 4).

## 5. Discussion

RS is a combination of micrognathia and glossoptosis that occurs with or without cleft palate [2]. It can occur as a single syndrome (IRS) or associated to other syndromes or anomalies (SRS) [4, 5]. Associated anomalies are common; the reported incidence varies from 26 to 82%, with 25 to 38% being syndromic cases [3, 21]. In the present study, 27% of RS infants presented associated anomalies or syndromes. The lower frequency of syndromes or other anomalies associated with RS observed herein may be due to the fact that we excluded infants who presented type 3 or 4 airway obstructions. Indeed, infants with type 3 or 4 airway obstruction usually have associated syndromes or other anomalies and tracheostomy is always required for respiratory release [4, 6].

The HRAC-USP is one of the few reference centers for cleft lip and palate in Brazil. Patients, mainly severe cases, come from distant hospitals from all over the country that lack specialized knowledge or adequate infrastructure to treat RS, which explains the high rate of severe cases and a wide age range of the patients treated at the HRAC-USP. Ideally, patients should be admitted to the HRAC-USP soon after birth, but, unfortunately, this does not usually happen in Brazil. However, in this study, most patients (62/107) were less than one month old at the time of their admission to the HRAC-USP.

The main clinical problems in RS infants are upper airway obstruction and feeding difficulties. Symptoms are highly heterogeneous, ranging from mild respiratory distress to severe asphyxia crises which become more frequent and more severe during the first months of life [4, 6]. In this series, all infants had upper airway obstruction with severe symptoms and feeding difficulties and only patients presenting type 1 and 2 airway obstruction were included. Type 1 was the most frequent, occurring in 89% of patients.

There have been many reports on different strategies to manage airway obstruction in patients with RS, but no consensus has been reached so far. Since the obstruction occurs at the base of the tongue, the treatments aim to move the tongue base forward, away from the airway. Interventions include prone positioning, NPI, glossopexy, tracheostomy, and osteogenesis mandibular distraction osteogenesis [6, 7, 9, 12, 13]. However, the lack of studies involving a large number of children with RS in a single center has hampered decision-making regarding the choice of treatment, especially whether or not surgical approaches should be used.

NPI has been used to relieve airway obstruction in patients with RS for over 25 years [7]. Because NPI improves breathing, this procedure also improves infants' ability to feed orally and, being an extremely simple procedure, can be performed at home by parents after being duly trained by the nursing staff during the infants' hospitalization [22]. Wagener et al. [23] reported successful outcomes in 20 children with RS. In their study, the children required NPI for 16–104 days but their entire time was spent in the hospital. Anderson et al. [24] reviewed the outcomes of home management of upper airway obstruction in RS using NPI and showed that treatment reduced in-patient stays and remained effective in home care. In addition, employing home management of RS patients, Abel et al. [17] successfully treated 63 of 77 patients with moderate and severe upper airway obstruction. In the present study, the total average time that the nasopharyngeal airway remained in situ was 57 days and the average hospital stay was 18 days, which is shorter than the average stay in similar studies [16, 22, 24]. The duration of NPI use in a hospital setting reported herein was similar to the results shown in previous studies [6, 8]. We also show that NPI was effectively and safely managed at home by trained parents, making early hospital discharge possible. All 107 patients were followed until NPI was no longer required and none of them needed tracheostomy, and they were decannulated with success.

Most infants (104/107) had cleft palate and all infants presented feeding difficulties. Generally, respiratory obstruction in infants with RS leads to difficulties in the coordination of suction and of swallowing and glossoptosis impairs anteriorization of the tongue which is necessary in order to obtain adequate suction. In addition, cleft palate creates a deficit in the negative intraoral pressure necessary to efficient suction, as well as inducing nasal reflux of food [20]. In this group of patients the improvement of respiratory difficulties with NPI led to improvement of feeding difficulties, and 85% of patients were fed orally. Fifteen percent underwent gastrostomy and the rate of gastrostomy was high in infants with SRS.

The natural history of patients with RS is an improvement with growth, for both the airway obstruction and feeding difficulties. Along with growth, airway obstruction improves as the mandibular growth and the coordination of the parapharyngeal muscles improves in conjunction with voluntary tongue control. The NPI improves breathing, allowing natural growth and resolution of airway obstruction without unnecessary surgical intervention.

The most relevant contribution of the present study is to present the NPI management protocol for children with RS adopted by the HRCA-USP, which was developed after extensive experience with these patients, and the fact that our results were obtained from a large number of patients from a single center. Indeed, NPI was the definitive treatment performed in all of the 107 infants studied here, corresponding to 48% of all patients with RS admitted to the HRAC-USP during the period studied. The children studied presented low morbidity and only 14% of them presented clinical complications in the first year of life. The main complication detected was pneumonia. Pulmonary complications and aspiration are the most severe and frequent complications of RS, generally due to deglutition disorders. The reported mortality varies from 2.6 to 30% [25, 26]. Indeed, Caouette-Laberge et al. [27] reported a 22.8% mortality rate in children with SRS and 5.9% for those with IRS. Importantly, no deaths occurred among the severe cases of RS treated exclusively with NPI using the HRAC-USP protocol.

We did not perform polysomnography (PSG), a sophisticated method to assess respiratory patterns and detect differences among infants not identified by oxygen saturation monitoring. The use of PSG could have improved the diagnostic accuracy in assessing the severity of upper airway obstruction (UAO). However, objective airway assessments using continuous oxygen saturation monitoring and clinical evaluation make the pediatric practice at the HRAC-USP and have been shown to be sufficient to diagnose the severity of airway obstruction and to detect clinical improvement [8, 9].

The HRAC-USP does not perform mandibular distraction osteogenesis during the neonatal period for the treatment of RS respiratory obstruction. Various studies have reported that mandibular distraction may avoid tracheostomy [28]. Mandibular distraction can help correct micrognathia by pulling the jaw forward, allowing the tongue to be pulled anteriorly via its anterior attachment to the mandible and thereby relieving airway obstruction. However, potential complications include inferior alveolar nerve damage, infections, dislodgment of distractor pins (causing injury to tooth buds), and anesthetic and surgical risks for newborns and young infants [27]. Thus, no consensus has been reached regarding the risks and benefits of this procedure for individuals with RS. Over the last few years, glossopexy surgery has been used less and less because the postsurgical results have been unsatisfactory in terms of airway release, especially in severe cases [29].

By studying a large series of infants with RS managed in a single center, we showed that NPI can be used to successfully treat these patients. The management of RS patients with NPI at the HRAC-USP involved a multidisciplinary and experienced team that was able to achieve a safe airway relief, low morbidity, and zero mortality rates for the infants treated.

## 6. Conclusions

NPI is an effective method for improving breathing and feeding in infants with RS and preventing surgical procedures in early infancy. The children treated with the NPI treatment protocol adopted at the HRAC-USP required a short hospitalization time and presented low morbidity and zero mortality during the first year of life.

## Figures and Tables

**Table 1:** Frequency of syndromes.

Syndrome	*N*	%
IRS	**78**	**73.00**
SRS		
Undefined syndrome	12	11.21
Stickler syndrome	9	8.41
Treacher Collins syndrome	4	3.70
Oculoauriculovertebral spectrum	1	0.92
Facial femoral syndrome	1	0.92
Otospondylomegaepiphyseal dysplasia (OSMED)	1	0.92
Otopalatodigital syndrome	1	0.92

Total	107	100.00

IRS: Isolated Robin Sequence.

SRS: Syndromic Robin Sequence.

**Table 2:** Type of respiratory obstruction in Isolated Robin Sequence and Syndromic Robin Sequence.

Syndrome	Type of respiratory obstruction					
	1		2		Total	
	*N*	%	*N*	%	*N*	%
IRS	71	91	7	9	78	100
SRS	24	83	5	7	29	100

Total	95	89	12	11	107	100

IRS: Isolated Robin Sequence.

SRS: Syndromic Robin Sequence.

**Table 3:** Gastrostomy in Isolated Robin Sequence and Syndromic Robin Sequence.

Syndrome	Gastrostomy					
	Yes		No		Total	
	*N*	%	*N*	%	*N*	%
IRS	7	9	71	91	78	100
SRS	9	31	20	69	29	100

Total	16	15	91	85	107	100

IRS: Isolated Robin Sequence.

SRS: Syndromic Robin Sequence.

**Table 4:** Morbidity rate in severe Robin Sequence managed with nasopharyngeal intubation during the first year of life.

Morbidity	IRS (*N* = 78)	SRS (*N* = 29)
Pneumonia	4	4
Bronchoaspiration	1	2
Apnea		1
Bronchospasm	1	
Gastrostomy other complications		1
Digestive hemorrhage	1	

Total	7 (8.97%)	8 (27.58%)

IRS: Isolated Robin Sequence.

SRS: Syndromic Robin Sequence.
